# Efficacy of a combination of esafoxolaner, eprinomectin and praziquantel (NexGard^®^ Combo) against *Thelazia callipaeda* in naturally infected cats[Fn FN1]

**DOI:** 10.1051/parasite/2024008

**Published:** 2024-02-28

**Authors:** Angela Di Cesare, Stefania Zanet, Donato Traversa, Mariasole Colombo, Eric Tielemans, Frederic Beugnet, Ezio Ferroglio

**Affiliations:** 1 Department of Veterinary Medicine, University of Teramo Località Piano d’Accio 64100 Teramo Italy; 2 Department of Veterinary Sciences, University of Turin Largo Paolo Braccini 2 10095 Grugliasco Turin Italy; 3 Boehringer Ingelheim Animal Health 29 Avenue Tony Garnier 69007 Lyon France

**Keywords:** Cat, Eprinomectin, NexGard^®^ Combo, *Thelazia callipaeda*

## Abstract

This clinical study assessed the efficacy of a topical combination of esafoxolaner, eprinomectin and praziquantel (NexGard^®^ Combo) in treating cats naturally infected with the eyeworm *Thelazia callipaeda* (Nematoda, Thelaziidae). On Study Day (SD) 0, sixteen client-owned cats with eyeworm infection were allocated to an untreated control group (G1, 8 cats) or to a NexGard^®^ Combo treated group (G2, 8 cats) and subjected to ocular examination. Cats in G2 received the treatment as per label recommendations. On SD 7 and 14 (±1), cats were examined for the presence of eyeworms and clinical signs. On SD 14, eyeworms were collected and counted. On SD 7 and 14, all cats in G1 were still infected with eyeworms, while G2 cats were free from eyeworms on SD 7 and 14, demonstrating 100% efficacy (*p* < 0.0001). All collected eyeworms were morphologically and molecularly confirmed to be *T. callipaeda*. On SD 0, fifteen out of the sixteen cats (7 in G1 and 8 in G2) displayed inflammatory ocular signs. On SD 7, all eight untreated cats and seven treated cats displayed inflammatory ocular signs. On SD 14, five out of eight G2 treated cats had recovered, while the eight untreated cats still displayed inflammatory ocular signs. The treatment significantly reduced lacrimation and conjunctivitis (*p* = 0.0001). No adverse reactions occurred. This clinical study provides evidence that NexGard^®^ Combo is highly safe and effective for the treatment of *T. callipaeda* infection in cats under field conditions.

## Abstract (fr)

Cette étude clinique a évalué l’efficacité d’une association topique d’esafoxolaner, d’éprinomectine et de praziquantel (NexGard^®^ Combo) dans le traitement des chats naturellement infectés par le ver oculaire *Thelazia callipaeda* (Nematoda, Thelaziidae). Au jour d’étude (JE) 0, seize chats appartenant à des clients et atteints d’une infection par le ver oculaire ont été attribués à un groupe témoin non traité (G1, 8 chats) ou à un groupe traité NexGard^®^ Combo (G2, 8 chats) et soumis à un examen oculaire. Les chats du groupe G2 ont reçu le traitement conformément aux recommandations de l’étiquette. Aux JE 7 et 14 (±1), les chats ont été examinés pour détecter la présence de vers oculaires et de signes cliniques. Au JE 14, les vers oculaires ont été collectés et comptés. Aux JE 7 et 14, tous les chats du G1 étaient toujours infectés par des vers oculaires, tandis que les chats du G2 étaient exempts de vers oculaires aux JE 7 et 14, démontrant une efficacité de 100 % (*p* < 0,0001). Tous les vers oculaires collectés ont été confirmés morphologiquement et moléculairement comme étant *T. callipaeda*. Au JE 0, quinze chats sur seize (7 en G1 et 8 en G2) présentaient des signes oculaires inflammatoires. Au JE 7, les huit chats non traités et les sept chats traités présentaient des signes oculaires inflammatoires. Au JE 14, cinq des huit chats traités par G2 s’étaient rétablis tandis que les huit chats non traités présentaient toujours des signes oculaires inflammatoires. Le traitement a réduit de manière significative le larmoiement et la conjonctivite (*p* = 0,0001). Aucun effet indésirable n’est survenu. Cette étude clinique indique que NexGard^®^ Combo est hautement sûr et efficace pour le traitement de l’infection à *T. callipaeda* chez les chats dans des conditions de terrain.

## Introduction

*Thelazia callipaeda* Railliet and Henry, 1910 (Spirurida, Thelaziidae) is a parasitic nematode causing ocular infection in a wide range of mammals, including cats, dogs, rabbits, and various wildlife species [1, 5]. This parasite may be of public health concern because it can also infect humans [5, 6, 26]. Its life cycle is indirect, involving definitive hosts (mammals) and intermediate vector hosts (fruit flies). In mammals, *T. callipaeda* inhabit the conjunctival sac and can also be found beneath the nictitating membrane. After mating, female worms release first-stage larvae (L1) in conjunctival secretions of the definitive host. The L1 are ingested by zoophilic male fruit flies when they feed on conjunctival fluids. Within the intermediate host, the L1 develop into the infective third-stage larvae (L3) [19, 21]. Transmission to a new vertebrate host takes place when male flies carry L3 infective stages and feed once again on the tear secretions of definitive hosts. In Europe, *Phortica variegata* (Drosophilidae, Steganinae) is the main fruit fly species acting as vector [1, 19, 21].

In the past decade, the parasite has spread in Europe, with reported cases of autochthonous transmission of *T. callipaeda* in pets in many European countries [5, 13, 23]. In Europe, cases of feline thelaziosis have been reported in Italy, France, Austria, Switzerland, Portugal, Bosnia and Herzegovina, Hungary, Greece, Romania [5], Germany [24], Spain [11], and Slovakia [13].

Clinical pictures of feline thelaziosis can vary in severity, ranging from subclinical cases to clinical signs such as tearing, eye scratching and inflammation, conjunctivitis, keratitis, blepharospasm, purulent exudations, and corneal ulcers [11, 28].

Timely and accurate diagnosis, followed by an effective therapeutic approach, is pivotal to safeguard animal health and reduce the circulation of *T. callipaeda* among intermediate and definitive hosts [28]. Nonetheless, there are limited treatment choices available for feline thelaziosis [14, 22], even though the confirmation of the efficacy of the macrocyclic lactone eprinomectin against the infection in cats is noteworthy [28].

The present study aimed to evaluate the safety and efficacy of the broad spectrum parasiticide formulation containing esafoxolaner, eprinomectin and praziquantel (NexGard^®^ Combo) against *T. callipaeda* in naturally infected cats under field conditions.

## Materials and methods

This clinical efficacy, negative controlled and blinded field study was conducted from February 2022 to May 2023, using a randomized block design based on order of inclusion. The ratio treated cats:untreated control was 1:1. The study was conducted in an area of northern Italy where *T. callipaeda* was previously observed in domestic and wild animals [18, 28]. Approval to conduct the study was obtained from the Italian regulatory authority (Italian Health Ministry, authorization no. 0004411-21/02/2022-DGSAF-MDS-A). The study was carried out on client-owned cats with written informed consent from the owner.

### Study design

On SD 0, sixteen privately owned healthy cats fulfilling the inclusion criteria were included. Inclusion criteria were: i) at least one live *T. callipaeda* worm in at least one eye, ii) ≥0.8 kg, iii) ≥8 weeks old, and iv) healthy (except for *T. callipaeda*-related clinical signs). Cats were excluded if they were: i) debilitated or suffering from disease or injury (other than eyeworm parasite infection), ii) fractious or otherwise unsuitable for inclusion, or iii) pregnant females or cats intended for breeding during the study.

After inclusion (SD 0), cats were randomly allocated to the untreated control group (Group 1 – G1) or to the NexGard^®^ Combo group (Group 2 – G2), treated on SD 0 according to the label instructions.

On SD 0, 7, and 14 (±1) cats were assessed for the presence of *T. callipaeda* by ocular examination and for clinical signs associated with thelaziosis. Specifically, bilateral ocular examination was carried out to assess the presence of clinical signs potentially related to thelaziosis and the infection level by *T. callipaeda* was categorized as: 0 worm, 1 worm, 2–5 worms, and >5 worms. No attempt was made to mechanically remove the eyeworms.

On SD 14, after ocular assessment, the conjunctival fornix of both eyes was flushed with ~5 mL of saline solution (0.9%) for confirmation of parasite presence/absence and recovery. *Thelazia callipaeda* parasites were removed, counted separately for each eye and stored in 70% ethanol for morphological and molecular identification [4, 17, 25]. Specifically, the molecular identification was performed by amplifying a portion of the mitochondrial cytochrome c oxidase subunit 1 gene (cox1, 689 bp) using primers NTF (5′-TGATTGGTGGTTTTGGTAA-3′) and NTR (5′-ATAAGTACGAGTATCAATATC-3′) [4, 20]. Amplicons were then sequenced and aligned, and sequences were compared with those in GenBank^®^. On SD 14, after the ocular assessment, G1 cats received a rescue treatment.

### Efficacy criteria

The primary criterion for the assessment of efficacy was the proportion of cats free of *T. callipaeda* worms on SD 14 in the treated group (G2) compared with the control group (G1). Cats were considered negative if no *T. callipaeda* worms were recovered on SD 14. The efficacy (%) was calculated at each time point using the following formula:

$$\mathrm{Efficacy}\;\left(\%\right)=(\mathrm{Proportion}\;\mathrm{of}\;\mathrm{positive}\;\mathrm{animals}\;\mathrm{in}\;\mathrm{the}\;\mathrm{untreated}\;\mathrm{group}-\mathrm{Proportion}\;\mathrm{of}\;\mathrm{positive}\;\mathrm{animals}\;\mathrm{in}\;\mathrm{the}\;\mathrm{treated}\;\mathrm{group})/\mathrm{Proportion}\;\mathrm{of}\;\mathrm{positive}\;\mathrm{animals}\;\mathrm{in}\;\mathrm{the}\;\mathrm{untreated}\;\mathrm{group}\times 100.$$

The second efficacy criterion for the assessment of curative efficacy was to compare the treatment groups with respect to the number of worms on SD 14 and/or presence of clinical signs caused by the eyeworm infection.

Counts were transformed to the natural logarithm of (count +1) for calculation of geometric means for each treatment group. Worm count reduction was calculated for each time-point using the following formula:

100×[(C-T)/C]

C: geometric mean among Control Group; T: geometric mean among the treated cats.

The differences between the groups were evaluated using a non-parametric Wilcoxon rank sum test.

All testing was two-sided at the significance level α = 0.05.

## Results

Sixteen client-owned cats (10 males and 6 females) from 7 months to 16 years of age and weighing 2.1 to 5.2 kg were included in the study. All cats lived outdoors or were allowed to free-roam. Both G1 and G2 consisted of 8 cats each. No concomitant treatments were necessary for any of the enrolled cats (based on standard criteria for thelaziosis management and pain relief), and no adverse reactions occurred during the study. All worms collected were identified as *T. callipaeda*. All samples produced amplicons of the expected size (689 bp). The sequence showed 100% homology with *T. callipaeda* mitochondrial partial COI gene for cytochrome oxidase subunit I, haplotype h1 (GenBank^®^ accession number AM042549.1).

### Parasitological efficacy

On SD 0, all cats were infected by *T. callipaeda*. On SD 7 and 14, the eight G2 cats were negative for eyeworm infection, while all cats in G1 were still infected (Table 1). Efficacy based on the proportion of cats free from eyeworm infection was 100% on both SD 7 and 14 (*p* < 0.0001, using a non-parametric Wilcoxon rank sum test) (Table 2).

**Table 1:** Individual level of *Thelazia callipaeda* infection on SD 0 and SD 7 and count after flushing on SD 14.

Group*	Case ID	SD 0		SD 7		SD 14		
		L	R	L	R	L	R	Total
1	101	1	1	1	1	1	1	2
	102	2–5	0	2–5	0	2	0	2
	105	1	1	1	1	1	1	2
	106	1	1	1	1	1	1	2
	109	0	1	0	1	0	1	1
	111	1	1	1	1	1	1	2
	112	1	1	1	1	1	1	2
	115	1	2–5	1	2–5	1	2	3
2	103	1	1	0	0	0	0	0
	104	1	0	0	0	0	0	0
	107	0	2–5	0	0	0	0	0
	108	2–5	2–5	0	0	0	0	0
	110	1	2–5	0	0	0	0	0
	113	2–5	1	0	0	0	0	0
	114	2–5	2–5	0	0	0	0	0
	116	1	0	0	0	0	0	0

L = Left eye; R = Right eye.

* Group 1 = Untreated control group; Group 2 = Treated with topical NexGard^®^ Combo.

**Table 2:** Efficacy based on the number of infected cats on SD 7 and 14. The difference between the groups was significant at *p* = 0.0001 on both SD 7 and SD 14, using a non-parametric Wilcoxon rank sum test.

Group*	SD 7	SD 14
1 (*n* = 8)	8	8
2 (*n* = 8)	0	0
Efficacy (%)	100.0	100.0

* Group 1 = Untreated control group; Group 2 = Treated with topical NexGard^®^ Combo.

On SD 14, G1 cats harbored an average of two worms per cats, compared to zero in G2 (100% efficacy). This difference is significant with a *p*-value of *p* < 0.0001 (non-parametric Wilcoxon sum rank test).

### Clinical efficacy

On SD 0, 15/16 (93.75%) cats (7 in G1 and 8 in G2) displayed inflammatory ocular signs (Table 3). On SD 7, all 8 untreated cats (G1) and 7/8 cats from G2 displayed inflammatory ocular signs. On SD 14, 5/8 cats from G2 had recovered, while the 8 cats from G1 still showed clinical signs. A statistically significant difference between G2 and G1 was observed for lacrimation and conjunctivitis with a χ^2^ test *p* of 0.0117 and 0.0005, respectively (*p* = 0.0001, using a non-parametric Wilcoxon rank sum test) on SD 14.

**Table 3:** Number (*n*/tot) and percentage (%) of cats showing one or more ocular signs compatible with thelaziosis on SD 0, 7, and 14.

	Group 1*			Group 2*		
	SD 0	SD 7	SD 14	SD 0	SD 7	SD 14
	*n*/tot (%)	*n*/tot (%)	*n*/tot (%)	*n*/tot (%)	*n*/tot (%)	*n*/tot (%)
Pruritus	5/8 (62.5)	4/8 (50)	4/8 (50)	1/8 (12.5)	0/8 (0)	0/8 (0)
Epiphora/Lacrimation	7/8 (87.5)	8/8 (100)	8/8 (100)	7/8 (87.5)	6/8 (75)	3/8 (37.5)
Purulent exudation	0/8 (0)	0/8 (0)	0/8 (0)	0/8 (0)	0/8 (0)	0/8 (0)
Conjunctivitis	6/8 (75)	7/8 (87.5)	7/8 (87.5)	7/8 (87.5)	6/8 (75)	1/8 (12.5)
Keratitis	0/8 (0)	0/8 (0)	0/8 (0)	0/8 (0)	0/8 (0)	0/8 (0)
Blepharospasm	2/8 (25)	2/8 (25)	2/8 (25)	2/8 (25)	0/8 (0)	0/8 (0)
Total number of cats showing ocular clinical signs	7/8 (87.5)	8/8 (100)	8/8 (100)	8/8 (100)	7/8 (87.5)	3/8 (37.5)

* Group: 1 = Untreated control group; Group 2 = NexGard^®^Combo-treated group.

## Discussion

This is the first clinical study assessing the curative efficacy of the topical combination containing esafoxolaner, eprinomectin and praziquantel (NexGard^®^ Combo) against *T. callipaeda* in naturally infected cats. This clinical study provides evidence that a single application of this product is effective and safe for eradicating eyeworms within 7 days after treatment. Furthermore, the results demonstrated a significant reduction of clinical signs caused by *T. callipaeda* infections after the administration of the product.

Feline thelaziosis may pose a significant challenge in feline veterinary practice due to nonspecific clinical signs (*e.g.*, conjunctivitis and epiphora/lacrimation) displayed by infected cats. These signs frequently overlap with those of more prevalent feline clinical conditions, including viral or bacterial infections. Hence, ocular signs often lead to the prescription of antibiotic or steroid-based eye drops without an accurate diagnosis. This practice is partly attributable to the reluctance to perform a comprehensive ophthalmic examination, a procedure that typically requires sedation or general anesthesia in cats [11, 15, 24].

To date, in Europe, the only licensed treatment option for feline thelaziosis is a spot-on formulation containing moxidectin/imidacloprid which showed an efficacy of 93.3% and 100% at 14- and 28-days post-treatment, respectively [22]. Other formulations have been evaluated off label in the treatment of *T. callipaeda* infections in cats. An oral product containing milbemycin oxime had an efficacy of 53.3% after a single treatment and of 73.3% after two treatments 2 weeks apart [14]. A spot-on formulation containing fipronil, (S)-methoprene, eprinomectin and praziquantel (Broadline^TM^) proved 100% efficacy within 2 weeks after a single treatment [28]. This latter product contains the same amount of eprinomectin and praziquantel as NexGard^®^ Combo and, accordingly, the results herein presented are consistent with what was observed in the previous efficacy study [28]. Of note, a single administration of NexGard^®^ Combo was effective at eliminating *T. callipaeda* infection within a shorter timeframe than previously reported for Broadline^TM^ (*i.e.*, 7 days *vs*. 14 days) [28].

From a clinical perspective, most treated cats showed a significant improvement of ocular signs, within 2 weeks of treatment. The persistence of clinical signs on SD 14 in some cats that were negative for *T. callipaeda* could be attributed to underlying chronic lesions, caused by the irritative action of the parasite striated cuticle, which may require more time for complete healing, as previously observed [16, 28]. Importantly, cats that still showed clinical signs at the end of the study had a high parasitic burden when enrolled.

The prompt resolution of the infection observed in the present study is not only beneficial for the affected cats but also for humans. In fact, these findings also have implications from a public health standpoint. Effective management of *T. callipaeda* in domestic cats minimizes the risk of zoonotic transmission to humans by reducing the occurrence of *T. callipaeda* among animal and human populations. This is relevant especially where *T. callipaeda* is endemic and poses a potential threat to both animals and people. Despite the fact that human thelaziosis is a rare disease, clinical cases have been described in several European countries (*e.g.*, Italy, Croatia, Germany, France, Spain, and Serbia) [5].

The monthly administration of macrocyclic lactones has proven effective in preventing thelaziosis in dogs [10, 12], while in cats, eprinomectin in combination with fipronil, (S)-methoprene, and praziquantel demonstrated high efficacy against the developing stages of lungworms and heartworms [2, 7, 8]. Therefore, the potential of eprinomectin in the prevention of the establishment of adult *Thelazia* spp. in cats warrants further investigation.

In conclusion, this study provides evidence of the clinical efficacy and safety of NexGard^®^ Combo against *T. callipaeda* in naturally infected cats. The observed reduction in clinical signs and clearance of the infection, coupled with the absence of adverse effects and the easy-to-apply spot-on formulation, underscores its potential suitability as a treatment option. The rapid and sustained efficacy demonstrated in this study further supports the potential of the product as a valuable tool in the management and control of parasitic infection in feline populations [3, 9, 27].

## Conflict of interest

The authors Angela Di Cesare, Stefania Zanet, Donato Traversa, Mariasole Colombo and Ezio Ferroglio declare that they have not conflict of interest. The authors Eric Tielemans and Frederic Beugnet, are employees of Boehringer Ingelheim Animal Health, Lyon, France, which sponsored the study.
